# Nanobodies: From High-Throughput Identification to Therapeutic Development

**DOI:** 10.1016/j.mcpro.2024.100865

**Published:** 2024-10-19

**Authors:** Peter C. Fridy, Michael P. Rout, Natalia E. Ketaren

**Affiliations:** Laboratory of Cellular and Structural Biology, The Rockefeller University, New York, New York, USA

**Keywords:** nanobody, single-domain antibody, monoclonal antibody, mass spectrometry, antibody display, antibody selection, antibody engineering, antibody therapeutics, V_H_H

## Abstract

The camelid single-domain antibody fragment, commonly referred to as a *nanobody*, achieves the targeting power of conventional monoclonal antibodies (mAbs) at only a fraction of their size. Isolated from camelid species (including llamas, alpacas, and camels), their small size at ∼15 kDa, low structural complexity, and high stability compared with conventional antibodies have propelled nanobody technology into the limelight of biologic development. Nanobodies are proving themselves to be a potent complement to traditional mAb therapies, showing success in the treatment of, for example, autoimmune diseases and cancer, and more recently as therapeutic options to treat infectious diseases caused by rapidly evolving biological targets such as the SARS-CoV-2 virus. This review highlights the benefits of applying a proteomic approach to identify diverse nanobody sequences against a single antigen. This proteomic approach coupled with conventional yeast/phage display methods enables the production of highly diverse repertoires of nanobodies able to bind the vast epitope landscape of an antigen, with epitope sampling surpassing that of mAbs. Additionally, we aim to highlight recent findings illuminating the structural attributes of nanobodies that make them particularly amenable to comprehensive antigen sampling and to synergistic activity—underscoring the powerful advantage of acquiring a large, diverse nanobody repertoire against a single antigen. Lastly, we highlight the efforts being made in the clinical development of nanobodies, which have great potential as powerful diagnostic reagents and treatment options, especially when targeting infectious disease agents.

## Highlights

• Successful methods used to produce high-affinity nanobodies, with a focus on proteomic approaches.
• Nanobody features enabling comprehensive sampling of an antigen’s surface.
• Advantages of a large diverse repertoire of nanobodies against an antigen.
• Nanobodies as promising therapeutics targeting infectious diseases, e.g.SARS-CoV-2.
• Key challenges when creating a nanobody-based therapeutic.

## Graphical Abstract

**Figure:**
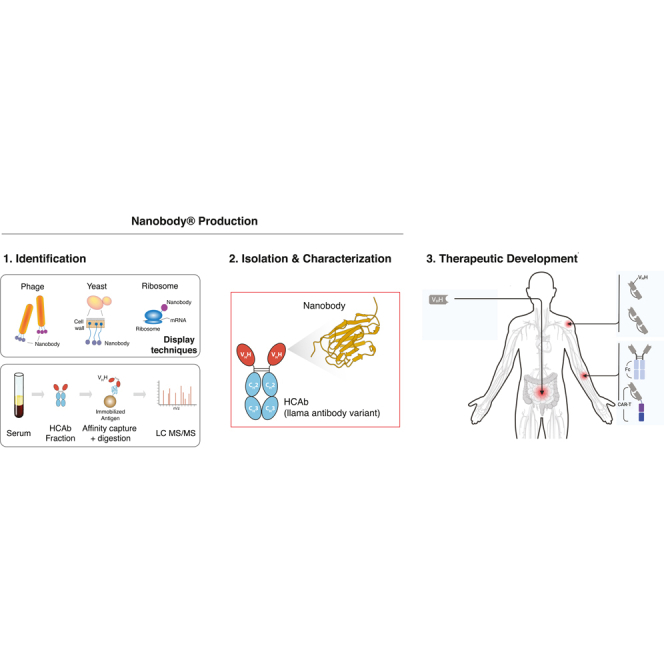

## In Brief

This review summarizes successful, widely used methods to produce high-affinity nanobodies against an antigen. We discuss the structural attributes of nanobodies that enable them to comprehensively sample their antigen surface and to engage in synergy pairs—underscoring the powerful advantages of acquiring a large, diverse nanobody repertoire against a single antigen. Lastly, we highlight the efforts being made in the development of nanobody therapeutics, which have great potential as diagnostic reagents and treatment options, especially when targeting infectious disease agents.

**Fig. 1:**
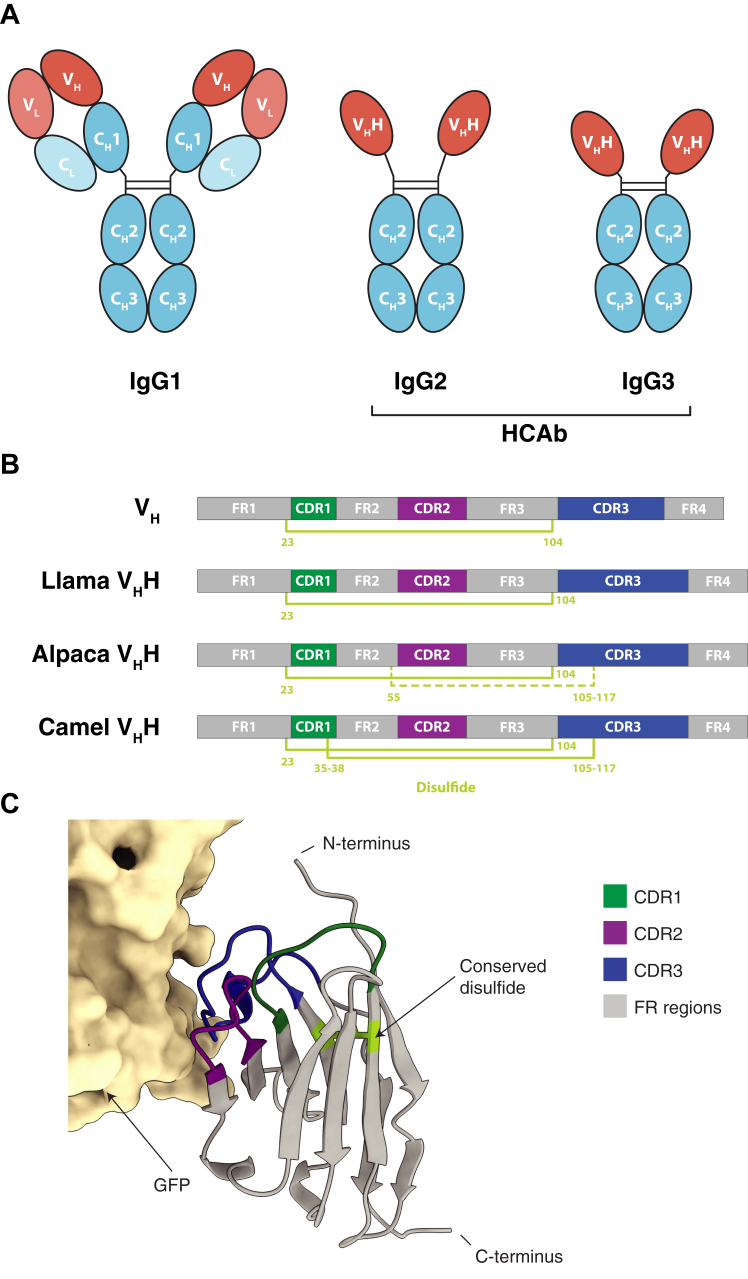
**Structural overview of nanobodies.***A*, diagram of HCAb and IgG domain structures. *B*, organization of typical V_H_H (nanobody) and V_H_ variable domains. CDR3 lengths vary substantially, but on average are higher in V_H_H domains from all species (averaging ∼14–18 *vs*. ∼11 residues in V_H_) (138, 139). In addition to the canonical disulfide (*yellow*) between cysteines at positions 23 and 104 in FR1 and FR3, camels commonly have a disulfide between CDR3 and position 35 to 38 in CDR1. At lower frequency (∼50%), alpacas have a cysteine at position 55 in FR2 that can form a disulfide with CDR3 (139, 140). Similar non-canonical cysteines and disulfides can also be found in llama V_H_H sequences, typically between CDR3 and CDR1 or FR2 residues, and more rarely in V_H_ sequences. Positions are numbered by IMGT convention. (CDR = complementarity-determining region; FR = framework). *C*, the crystal structure of an anti-green fluorescent protein (GFP) nanobody (*gray*) bound to GFP (*sand*) (PDB ID: 8SG3), highlighting the three CDR and FR regions.

The precision targeting power of antibodies makes them some of the most widely utilized biologic reagents in both general scientific research and therapeutic development (1, 2, 3). Antibody-based therapeutics have provided a significant advance in the fight against numerous human diseases, especially cancer (4, 5, 6, 7). A compelling single domain antibody fragment, termed *nanobody* (a registered trademark of Ablynx NV, Sanofi) has emerged as an alternative to the precision targeting power of conventional monoclonal antibodies (mAbs), with several advantages over mAbs whilst filling complementary roles (8, 9). Generated from the variable domain of a unique subset of immunoglobulins found in camelids (*e.g.* llamas and alpacas), nanobodies consist only of heavy-chain homodimers, with no associated light chains (Fig. 1), and at only ∼15 kDa they are the smallest known antigen-binding single polypeptide chain occurring in any natural antibody (10, 11). Due to their small size, nanobodies can target epitopes inaccessible to conventional antibodies, and coupled with their ease of production (especially when compared with other mAbs), they can make ideal research tools with myriad applications from crystallographic chaperones to diagnostic development (8).

The application of nanobodies in scientific research is well-established and well-covered elsewhere (8, 12, 13, 14). In recent years, it is in therapeutic and diagnostic development that nanobodies have received considerable attention. To date, numerous nanobody therapeutics have entered the global therapeutic market, beginning with Caplacizumab, a bivalent nanobody drug that received global approval for use in 2019 for the treatment of the autoimmune disease aTTP (acquired thrombotic thrombocytopenic purpura) (15, 16). Following the success of Caplacizumab, other nanobody-based therapeutics have been approved for use in various countries, including Ozoralizumab in Japan, for treatment of rheumatoid arthritis (17, 18), the cancer immunotherapy Envafolimab in China (19, 20), and the nanobody-based CAR-T therapeutic Ciltacabtagene autoleucel, with both FDA and EU approval (21). Infectious disease research has also seen significant interest in the therapeutic potential of nanobodies, including efforts to combat multi-drug resistant bacteria (22, 23) and rapidly evolving human viruses (24, 25, 26). This review aims to summarize the benefits in generating large, diverse repertoires of nanobodies against a single antigen, the different approaches used to achieve this, with a focus (as befitting this journal) on more recent mass spectrometry (MS)-based proteomic techniques, and to highlight promising applications of nanobody technology, especially for the treatment and diagnosis of infectious diseases.

## Techniques for Nanobody Discovery

**Fig. 2:**
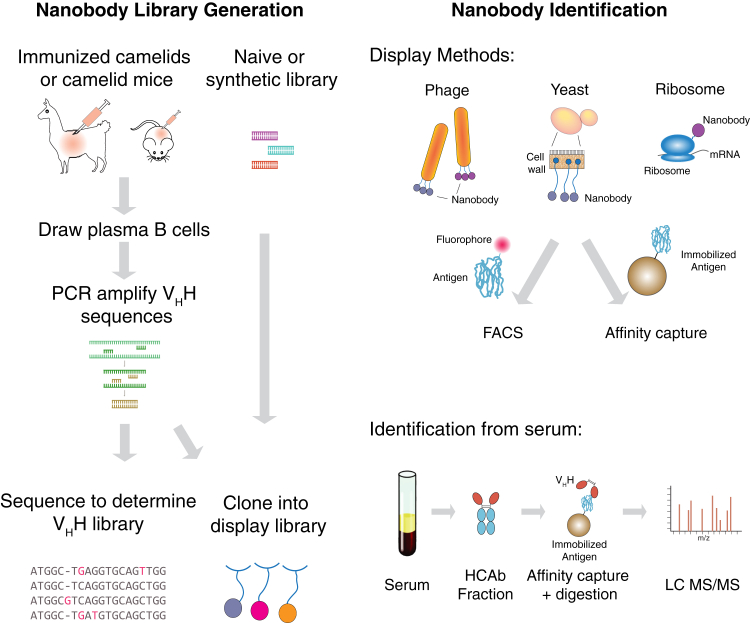
**Outline of methods used to identify antigen-specific nanobody candidates.** Libraries of V_H_H (nanobody) DNA sequences can be produced from camelids (llamas, alpacas, camels) immunized with antigen(s) of interest, or mice genetically engineered with germline V_H_H. These can be directly sequenced or cloned into a display library, or libraries can be prepared from naïve or synthetic libraries independent of immunization. Libraries can then be screened in a variety of systems, including phage display, yeast display, or ribosome display. Antigen-binding clones can be enriched by affinity capture, or by FACS after incubating with fluorescently-labeled antigen. Binding antibodies can alternatively be identified directly from serum, with the HCAb fraction affinity-purified, and antigen-bound nanobody fragments analyzed by LC-MS/MS to identify nanobodies from an *in silico* sequence library.

A variety of alternative approaches to generating nanobodies have emerged over recent years; all underscore the relative ease of nanobody production over conventional mAbs. These techniques all start with a large, diverse collection of nanobody clones, ultimately derived from camelid immune cells (usually from llamas and alpacas, and less commonly, camels). Nanobodies against a target of interest are then selected by a range of methods: through expression display and panning of an intermediate clonal library (phage, yeast, or ribosome display), or by direct detection of relevant serum-derived antibodies (MS-based identification) (Fig. 2). The key step in all of these techniques is identifying sequences of nanobodies (the antigen-binding V_H_H variable domains) from target-specific heavy chain-only antibodies (HCAbs) (Fig. 1). Libraries used in these techniques are typically prepared from camelids immunized with the target antigen of interest, and for savings in cost and time, mixtures of at least 5 to 10 antigens can be used in immunizations with no loss in immune response strength (27, 28). As animal husbandry for camelids can be demanding and require outsourcing, mice have been engineered with germline camelid nanobody sequences that can mature HCAb antibodies in response to immunization as a more laboratory-accessible option, though the overall immune diversity of these mice is significantly lower than camelids (29).

There has been increasing interest in the use of naïve libraries for nanobody screening, in which sufficiently large and diverse libraries from non-immunized animals, expanded using synthetic techniques, or fully synthetic libraries, can be panned for antigen-specific HCAbs (30, 31, 32, 33, 34). Publicly or commercially available libraries have made this a particularly accessible option for investigators by eliminating the need for animal work. While this saves considerable time and resources on animal immunization, starting affinities of binders identified from naïve libraries tend to be lower, typically requiring affinity maturation or multimerization to approach the sub-nanomolar affinities routinely achieved by immune-raised nanobodies (31).

### Display Library Approaches

Display library screening has been the most common technology for nanobody selection, providing the majority of nanobodies in use. Phage display was the first method applied to identifying nanobodies from camelids and continues to be used routinely, though yeast display and ribosome display are now in wide use as well. Typical display approaches rely on cloning libraries from nanobody sequences amplified from lymphocyte cDNA for surface expression in phage (35, 36, 37) or yeast (33, 38, 39), and panning against the antigen of interest. This is a widely available approach that has been successfully applied to a multitude of antigens, and remains the most accessible approach in nanobody development. While both yeast and phage libraries have proven effective, yeast display offers advantages by allowing for direct selection of clones by cell sorting, and even release of soluble protein from the cell surface for analysis (39). Recent improvements in this method have made it more robust, with optimizations to surface fusion protein design, library preparation, and panning parameters (33, 39, 40).

Ribosome display is an alternative and cell-free screening approach, in which library mRNA is translated *in vitro* but protein is prevented from leaving the ribosome, to preserve a complex with both a nanobody protein and its encoding transcript that can then be screened against antigens and sequenced (41, 42). This fully *in vitro* approach allows for larger libraries than cell-based methods (10^10^-10^12^), making it particularly useful for naïve libraries (see below) (34, 43). This technique has also been incorporated into Illumina high-throughput sequencing platforms, allowing *in situ* sequencing and measurement of antigen binding activity of up to ∼10^8^ clones (44). While this technique is subject to the limitations of *in vitro* translation and naïve libraries (above), it provides a highly parallelized alternative to nanobody screening, with possibilities for additional functional assays on immobilized libraries.

### Mass Spectrometry in Nanobody Identification

The introduction of proteomic, mass spectrometry-based methods for nanobody identification has provided a complementary approach to display procedures (45, 46, 47, 48, 49). With this technique, lymphocyte cDNA is again prepared from immunized animals, but rather than cloning into a display library, PCR-amplified nanobody sequences are directly analyzed by next-gen sequencing to generate an *in silico* library of HCAb variable domains. To identify antigen-specific sequences, HCAbs from sera are affinity-purified against the target antigen, optionally digested to remove Fc, and bound nanobody fragments analyzed by LC-MS/MS. Identified peptides are then matched to the *in silico* nanobody library to determine full-length sequences to be synthesized and expressed as nanobody candidates. This approach has the advantage of directly analyzing antibodies in serum, allowing correlation of nanobody sequences to the animal’s effective immune response. The use of protein rather than cell-based affinity isolation also allows significantly more flexibility in this stage of selection, as there is no need to maintain cell viability for a display library. This allows for significantly higher stringency in wash conditions (*e.g.* salt, detergent, pH), facilitating enrichment of ultra-high-affinity binders, or antibodies able to retain stability and binding under a given biochemical condition.

However, MS analysis of purified HCAb samples also presents unique challenges compared to typical proteomic samples. These concerns are broadly shared with antibody proteomics in general, though significantly simplified by the lack of a light chain (50). First, antibody repertoires are highly divergent between individuals, meaning that sequence libraries are needed for each immunized animal. This can be addressed by sampling B cells from animals post-immunization for HCAb sequencing from cDNA. The need for animal-specific libraries can be alleviated to some extent by combining many antigens for a single immunization round.

**Fig. 3:**
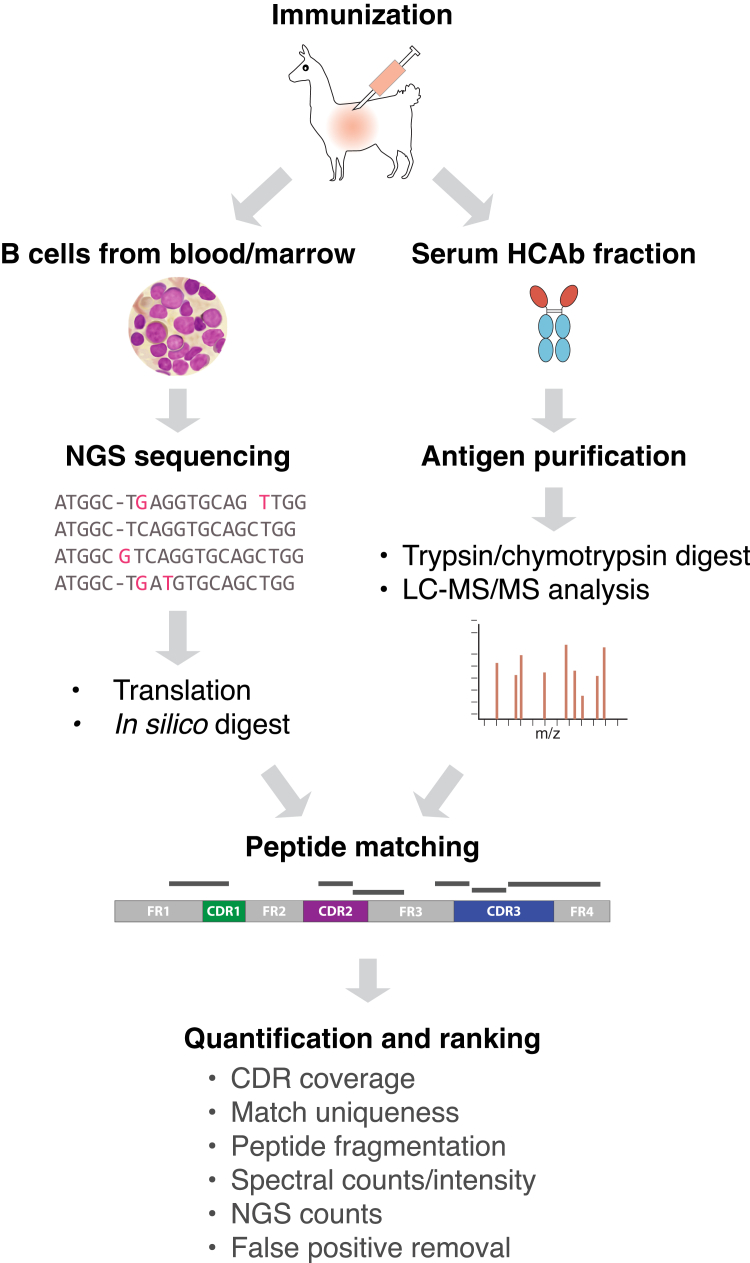
**Overview of a typical mass spectrometry approach to nanobody identification**. After camelid immunization, both sera and lymphocytes from blood or marrow are sampled from the animal. A sequence library of all nanobody-expressing transcripts is generated by next-gen DNA sequencing and prepared for peptide searching by *in silico* translation and protease digestion. In parallel, an HCAb fraction is prepared from serum and affinity-purified against the target antigen. Purified nanobody-encoding HCAb fragments are separated by SDS-PAGE and digested by trypsin and/or chymotrypsin before LC-MS/MS analysis. MS data is then searched against the corresponding animal-generated sequence library, and peptide matches used to identify high-confidence nanobody clones by algorithmic weighting of coverage and other parameters.

Second, the high sequence similarity of framework regions within the variable domain necessitates specialized processing to unambiguously identify unique nanobody sequences; the nanobody-defining complementarity-determining regions (CDRs), the essential antigen-binding loops of all antibodies, must therefore have adequate peptide coverage for sequence matching, in particular CDR3, generally the longest, most diverse, and most relevant to binding (51). Multiple groups have utilized similar approaches using bottom-up MS to facilitate such analyses (Fig. 3) (45, 46, 49). Key features of these pipelines include customized software used to search large databases of nanobody sequences, and matching and validating peptides covering CDRs to identify high-confidence nanobody candidates. High sequence conservation, as well as the potential for non-specific HCAb contaminants, makes false positives a particular concern in this analysis, and these pipelines are largely reliant on unique fingerprint peptides. One way to reduce these false positives is by using a decoy database to identify and filter out non-specific nanobody sequences, either from a separate non-immunized animal or if possible from a pre-immune library of the same target animal (46).

In addition to explicit filtering using decoy data, optimized searching and sequence ranking algorithms are key to the confident identification of antigen-specific nanobody sequences. Peptide coverage of variable CDR regions must be appropriately weighted, as these are the key determinant fingerprints of unique sequences. In particular, peptide fragmentation across CDRs must be assessed to confirm true matches, as framework sequence alignment alone is not sufficient for unique identification. Furthermore, the amino acid distribution in and around the key CDR3 region tends to limit coverage by tryptic peptides; chymotrypsin has been found to more reliably cover this area, and thus a combination of proteases is preferred for optimal sequence coverage (46, 49).

### Comparative Advantages and Applications

MS-based nanobody identification is highly complementary to display library screening, each with unique advantages and disadvantages. MS can be biased against sequences that do not readily provide detectable or identifying peptides, while display approaches can select against rare clones in the library or those that are poor expressers or binders in the context of the surface display system. Differences in sensitivity to binding affinity may also affect results between these approaches, as display methods with multiple antibody copies will have a greater avidity effect (higher effective affinity due to multiple binding interactions) than single-molecule serum IgG isolation. Notwithstanding these differences, both approaches have been applied with great success against diverse antigens, and can even be used in combination to increase overall nanobody coverage and diversity (40, 47). While it is difficult to directly compare the results of these techniques due to the large number of variables in antigen immunogenicity, animal responses, and repertoire sizes, in one example targeting SARS-CoV-2 spike with identical source libraries using either yeast display or MS-based identification, similarly high hit rates and affinities were obtained up to at least 30 candidates, though greater overall diversity allowed larger numbers of unique candidates from MS identification in this case (40, 47).

## The Benefits of Large Repertoires of Nanobodies

As discussed above, an MS approach enables the identification of high-confidence nanobody sequences from the immune response of the animal (47), where the uniqueness of the CDR3 region is first identified and further used to group the repertoire of potential nanobodies based on CDR3 sequence similarity. Mast *et al*. (47) revealed the successful application of this method when generating nanobodies against the full-length spike protein from ancestral SARS-CoV-2. They identified 374 high-confidence nanobody sequences, which were further categorized *via* their CDR3 sequences into 183 groups or “nodes.” They observed that nanobodies within each node displayed similar antigen binding behavior, to suggest more similar epitope coverage within each node than between each node. The authors then functionally characterized nanobodies from each major node, which enabled them to generate a final repertoire of 116 high-affinity nanobodies, importantly targeting all major domains of the spike protein. The repertoire of nanobodies identified by Mast *et al*. (47) revealed numerous nanobody unique (“hidden”) epitopes on spike, uncharted by mAbs targeting the same antigen (47, 52).

**Fig. 4:**
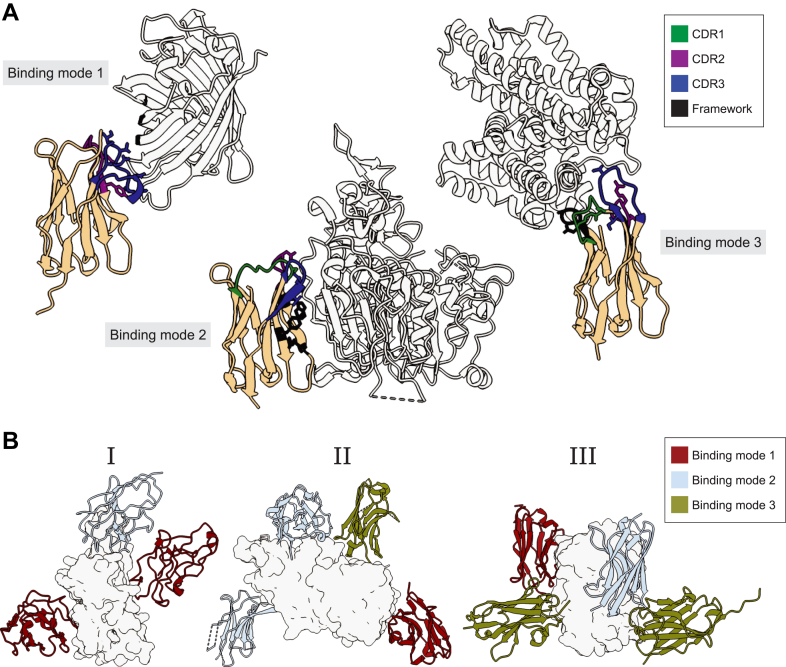
**Nanobodies bind antigens in diverse orientations.***A*, the three different orientations (Binding mode 1, Binding mode 2 and Binding mode 3) a nanobody can bind its antigen as determined by the composition of its paratope. *B*, nanobodies which bind their antigen in one of the three binding modes as defined in (*A*) are shown to be able to bind simultaneously to their target antigen. Panel I – three different anti-RBD nanobodies bound to RBD (59); Panel II – four different nanobodies bound to P domain of norovirus (58); and Panel III – four different anti-GFP nanobodies bound to GFP (53).

The small size of nanobodies is believed to be key to accessing these “hidden” epitopes on antigens. Additionally, numerous studies have collated structural information from the protein data bank (PDB) to demonstrate that nanobodies can bind their antigen in different orientations (often utilizing their framework (FR) regions to do so), greatly aiding in comprehensive sampling of their antigen surface to access and bind a variety of epitope shapes (53, 54, 55, 56). This is in stark contrast to conventional mAbs which interact primarily *via* their three CDRs, in what can be described as a “head-on” manner (10, 53). This comparative freedom of interaction orientation and ability to utilize large portions of the FR regions enables nanobodies to create unique compositions of epitope binding surfaces (paratopes) when bound to their antigen, such as seen with anti-GFP nanobodies (53, 57), anti-norovirus capsid nanobodies (58), and anti-SARS-CoV-2 RBD nanobodies (53, 59) (Fig. 4*A*). These different orientations have numerous benefits, as they potentially aid in: (i) comprehensive sampling of the antigen surface (Fig. 4*B*); (ii) attacking numerous distinct epitopes on an antigen and (iii) enabling multiple nanobodies to bind the same antigen simultaneously, an ability greatly aided by their size.

The ability of multiple nanobodies to bind a single antigen simultaneously is especially useful when applying nanobody technology to the generation of therapeutics and diagnostics, particularly against antigens of rapidly evolving disease agents. This was exemplified by Mast *et al*. (47), who showed how neutralizing nanobodies binding distinct epitopes on the receptor binding domain (RBD) of SARS-CoV-2 spike readily engaged in synergy, in which nanobody pairs produced neutralization mixtures far more potent than the predicted additive behavior of the nanobodies’ separate activities (47). The authors showcased numerous synergistic relationships between different nanobody pairs selected from their nanobody repertoire, many of which remained effective against numerous SARS-CoV-2 strains (47, 52). In addition, factoring in the different orientations different nanobodies adopt when binding an antigen can greatly facilitate the rational design of multivalent formulations of nanobodies as research tools and therapeutics, where multimeric formatting is a common feature of the nanobody therapeutics currently licensed and in clinical trials (60).

## Applying Nanobodies in Proteomic Research

Researchers have utilized the unique advantages of nanobodies in a wide variety of research and clinical applications, including microscopy and structural biology, which have been extensively surveyed (8, 9, 61, 62).

To highlight one expanding area of use, nanobodies’ size and ease of modification have made them particularly suited to various approaches in proteomic research. Several nanobody toolkits have been implemented for the purification of protein complexes for *ex vivo* proteomic and structural studies, by targeting widespread tags such as GFP or novel peptide tags (45, 57, 63, 64). While retaining the high affinity and specificity of traditional antibody immunoprecipitation, nanobodies offer further flexibility in ease and low cost of production. The resilience and small size of nanobodies also make them more amenable to on-bead structural analysis of captured complexes, for example by cross-linking MS or cryo-EM (65, 66). Nanobodies can also be compatible with *in vivo* expression in heterologous systems and have been extensively used in intracellular constructs or “intrabodies” to explore protein localization and for proteomic and interactomic studies. Nanobodies can be expressed either independently or as genetic fusions to selectively target a tagged or native antigen for functional regulation, visualization, or biosensing (67). Intracellular nanobodies can also be used for targeted protein modifications, such as biotin-labeling *via* fusions with biotin ligase (BioID or TurboID), enabling proximity-based mapping of protein-protein interactions. This adaptable technique has been applied with nanobodies targeting GFP-tagged proteins in zebrafish and *Caenorhabditis elegans* (68, 69). Developing nanobodies for intracellular use can be a challenge, as many nanobodies do not fold properly in the cytoplasm, primarily due to the lack of required disulfide formation in the reducing intracellular environment, demanding either selection or engineering of suitable clones. Many approaches have been developed to engineer suitable nanobodies (surveyed in (67)) including cloning CDRs into optimized scaffolds (32), directly selecting candidates for intracellular activity in two-hybrid or other functional screens (70, 71), or sequence engineering by mutations in key residues (72).

Several nanobody-tag combinations have also been characterized to facilitate *in vivo* use of nanobodies. These include 10 to 14 amino acid “NanoTag” peptide tags that were developed with corresponding nanobodies and validated in *Drosophila* (73), the 14 amino acid ALFA-tag and its high-affinity NbALFA nanobody partner (63), and others (74). These short peptide tags are more easily incorporated into target proteins without disrupting function, making them well-suited to studies using nanobodies against intracellular targets, and can be used in combination (64, 75).

## Nanobodies Against SARS-CoV-2 and Infectious Diseases

Nanobodies are increasingly being applied as reagents for the treatment and diagnosis of infectious diseases. Currently, two nanobodies, ARP1 and LMN-101, are in clinical trials for the treatment of diarrheal disease caused by rotavirus (76) and *Campylobacter jejuni* (77) respectively. Additionally, nanobodies are being explored as a treatment option for placental malaria by targeting the VAR2CSA protein of *Plasmidum falciparum* (78); influenza by targeting the M2 surface protein of the virus (79) and also conserved regions on Hemagglutinin (HA) (80); human papilloma virus (HPV) (81, 82, 83) and human African trypanosomal disease (84). As mentioned previously, the ability of nanobodies to target non-immunodominant regions on proteins, which are often highly conserved, establishes the opportunity of generating broad-spectrum nanobody therapeutics against a pathogen. This is exemplified by the nanobodies targeting influenza virus, where nanobody M2-7A and several other nanobodies successfully targeted the highly conserved ion channel and regions of HA on the viral surface (79, 85, 86). This suggests the possibility of formulating an influenza treatment effective against multiple influenza strains.

In recent years, SARS-CoV-2 has been a prime testbed for therapeutic solutions to a rapidly evolving disease agent, with many efforts demonstrating the unique properties of nanobodies. The COVID-19 pandemic saw a dramatic rise in the development of nanobodies as both therapeutics and diagnostics against the SARS-CoV-2 virus, targeting the viral spike protein. Numerous SARS-CoV-2 spike-neutralizing nanobodies have been identified, binding spike epitopes distinct from those bound by mAbs (47, 59, 87, 88). In the work by Mast *et al*. (47), their repertoire of over 100 high-affinity nanobodies targeted all major regions of the SARS-CoV-2 spike, with many nanobodies binding highly conserved regions hidden from the selective pressure of the conventional mAb response. This included the highly glycosylated S2 domain and regions on the RBD resistant to mutational escape. Several of these regions showed strong conservation between SARS-CoV-2 variants, resulting in the identification of pan-variant nanobodies that remained effective at neutralizing emerging strains of the virus (52, 59, 87), in contrast to conventional mAbs that began to lose viral neutralizing efficacy as new strains of the virus rapidly emerged (89). Furthermore, Ketaren *et al*. (52), showed how nanobodies within their repertoire were specific for certain variants of SARS-CoV-2, which introduces the possibility of creating diagnostic agents able to discriminate between different viral strains, enabling a tailored approach to treatment upon infection.

Unsurprisingly, efforts are being made to develop broad-spectrum nanobody-based therapeutics against betacoronaviruses by targeting highly conserved regions on spike, where a single nanobody could potentially target multiple members of the betacoronaviral family. This is exemplified by Laroche *et al*. (90), where the authors used deep mutational scanning to design nanobodies able to target both SARS-CoV-1 and SARS-CoV-2. Furthermore, as mentioned earlier, the ability of two nanobodies to readily engage in synergy to create potent neutralization mixtures against the ancestral SARS-CoV-2 virus has recently been shown to extend the effectiveness of the nanobody repertoire established by Mast *et al*. (47) in neutralizing SARS-CoV-2 variants from successive new strains (52). Intriguingly, the authors revealed a unique synergy relationship between two nanobodies, where the combination of a neutralizing and non-neutralizing nanobody targeting the Delta variant of SARS-CoV-2 still exhibited synergy to create a potent synergistic cocktail. This flexible capacity for synergistic behavior opens up a potentially powerful property of nanobodies to consider when designing nanobody therapeutics, which is enabled by a repertoire of nanobodies large enough to contain binders with numerous non-overlapping epitopes. Aside from the synergy observed between nanobodies targeting spike of SARS-CoV-2 (47, 52), nanobody synergy mixtures have also been observed between nanobodies developed against the capsid of norovirus (91) and HPV nanobodies (82). With the increasing use of nanobody technology to target human disease agents, especially with pipelines (as described in this review) available that maximize nanobody sequence identification to generate highly diverse nanobody repertoires, the compelling property of synergy is sure to become a powerful feature when utilizing nanobodies in both a research and clinical setting.

Collectively, the large body of work accumulated since the onset of the COVID-19 pandemic in generating nanobodies against the spike protein of SARS-CoV-2 has exploited many unique facets of nanobodies, drawing particular attention to the power of their much smaller size to overcome many limitations of conventional mAbs when developing persistent therapeutic options against infectious disease agents. Additionally, the rapid response of researchers during the COVID-19 pandemic who engaged nanobody technology to provide both nanobody tools and strategies to combat the virus, reveals the enormous potential to apply this technology to other viruses, particularly those that may have a high pandemic potential.

## Considerations When Developing Nanobodies as Therapeutics

**Fig. 5:**
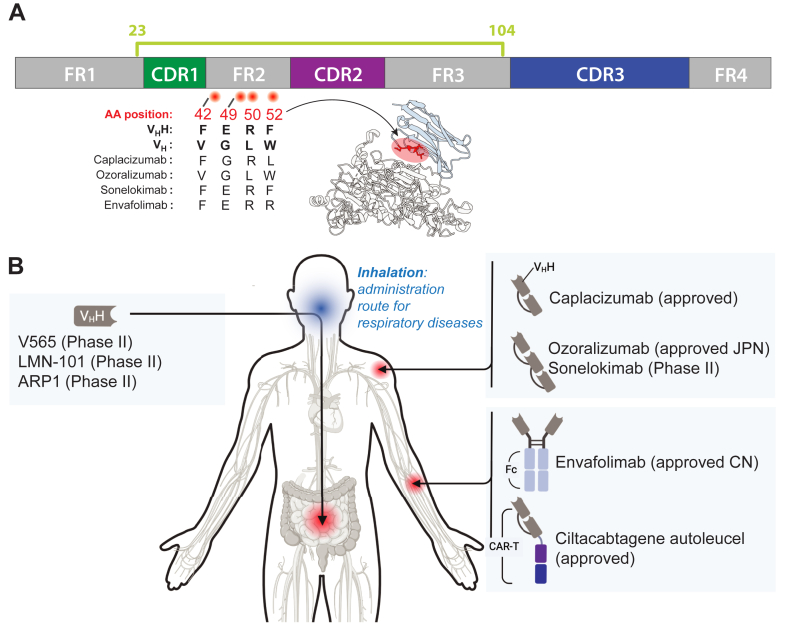
**Humanization and therapeutic applications of nanobodies.***A*, linear schematic of a typical nanobody structure. Regions on the structure commonly humanized, yet that have been shown to participate in antigen binding, are represented as red circles with the corresponding sequence numbering (IMGT) (Adapted from a meta-analysis of ∼100 nanobody-antigen interactions analyzed from the protein data bank (PDB) (53)). The amino acid substitutions within FR2 for the corresponding drugs are annotated (determined from the sequences of each drug sourced from https://gsrs.ncats.nih.gov/ginas/app/beta/home or 7EOW for Caplacizumab). The potential effect on binding when targeting these four residues on nanobodies that adopt Binding mode 2 (PBD ID: 5my6) is shown. *B*, different strategies to deliver and create approved nanobody therapeutics. Figure created with BioRender.com. CN, China; JPN, Japan.

Critical design elements when developing a nanobody therapeutic include (i) nanobody humanization to limit potential immunogenicity; (ii) the final drug structure and oligomeric state, which often requires optimization to prevent overly rapid systemic clearance of nanobodies and/or improve antigen binding (92), and (iii) the drug administration route, which coupled to (ii) influences the bioavailability of the drug (Fig. 5).

### Humanization

Mechanisms to humanize nanobodies are covered extensively elsewhere (9, 93, 94) and largely involve mutating residues within the nanobody scaffold (FR region) to better mimic that of the human V_H_ domain, particularly that of the FR2 region that is a main point of difference between camelid V_H_H and human V_H_ (94). (Fig. 5*A*). Though nanobody humanization is commonly practiced, there are conflicting interpretations as to whether humanization in fact reduces immunogenicity or limits anti-drug antibodies (ADAs) targeting the nanobody therapeutic. A study performed by Ackaert *et al*. (95) tested non-humanized nanobodies targeting human epidermal growth factor receptor 2 (HER2) and macrophage mannose receptor (MMR) in an *in vitro* immunogenicity assay, and saw no significant induction of a T-cell response to suggest strong immunogenicity. Though many humanized nanobody drugs tested clinically have similarly shown minimal immunogenicity (93, 96), this is not always the case – for example, one humanized tetravalent nanobody targeting the TNF death receptor 5 (DR5), resulted in hepatotoxicity and detection of ADAs targeting the nanobody-drug, leading to termination of the study (97). These results suggest that immunogenicity is not limited to the presence of non-human sequences on the protein-based therapeutic, and the contribution of other factors such as pre-existing ADAs and instability of the therapeutic must also be considered (97, 98). Furthermore, it is important to note that the presence of ADAs has been shown to be a common characteristic of many approved protein-based therapeutics, including human monoclonal antibodies (99, 100). Ultimately, with humanization a feature of all approved nanobody-based drugs (Fig. 5*A*), it remains a measure commonly pursued when developing nanobodies for human use.

The strategies that are employed to humanize nanobodies largely involve engineering the FR regions, particularly FR2, which, as shown above, can impact antigen binding and/or the biophysical properties of nanobodies (53, 94). This is seen when mutating the FR2 region, which is shown in numerous structures to be involved in both antigen binding (53) and maintaining nanobody solubility (94). As a nanobody’s binding and biophysical properties can be impacted by humanization, one way to circumvent the impact of classical humanization strategies on nanobody binding would be to select for nanobodies within a repertoire that did not utilize key scaffold regions for binding—underscoring the value of having a large, diverse starting repertoire of nanobodies. With the growing number of high-resolution nanobody-antigen structures, it is increasingly likely that computational modeling can be used to predict what residues are involved in antigen binding (*i.e.* predict the nanobody paratope). For example, FR2 is often recruited to bind an antigen in nanobodies with a short CDR3 (53, 101) – this information could be used to predict nanobodies that use FR2 to bind their antigen, preventing issues with humanization. Furthermore, to aid in the rational design of humanized nanobody therapeutics, computational programs such as Llamanade (102) and AbNativ (103) have been developed to guide strategies to humanize nanobodies, potentially without compromising both solubility and binding, as recently reviewed in Gordon *et al*. (96).

### Post-Translational Modifications

A major concern for protein therapeutics, and particularly antibodies, is enzymatic or chemical modification during production or storage. Post-translational modifications (PTMs) such as glycosylation, deamidation, and oxidation can affect therapeutic efficacy and bioavailability, and controlling and identifying such changes is a major challenge in biologic drug development (104, 105, 106). Nanobody-based therapeutics share many of these challenges as protein products, but the smaller and simpler structure relative to mAbs can make them more amenable to production, optimization and analysis. Modifications during cellular expression can be a significant challenge for production of molecularly homogeneous conventional antibodies, but are generally not a concern for nanobodies—they are typically produced recombinantly in bacteria, avoiding enzymatic glycosylation or modification otherwise seen with antibodies. When eukaryotic production is required, the nanobody’s single domain lacks canonical sites of antibody modification (*i.e.* the F_c_ region) (107), and can be engineered as needed to avoid spurious post-translational modification sites as these are not normally required for activity.

With their smaller size, nanobodies are also more amenable to structural analysis than standard antibodies, particularly by mass spectrometry. At ∼15 kDa, nanobodies are small enough for direct analysis in top-down mass spectrometry (TD-MS) (108, 109, 110), allowing for detection of modifications, and avoiding the need for middle-down methods (pre-cleavage of antibody fragments) or other customized techniques typically required for intact MS of 10× larger mAbs (111, 112). Effective direct fragmentation of nanobodies for sequence assessment has also been demonstrated with TD-MS, in one case showing sequence coverage of 87% from a combination of HCD, ETD, and ultraviolet photodissociation (UVPD) (110). As with standard antibodies, bottom-up MS can also be applied for detection of modifications, with a combination of trypsin and chymotrypsin particularly effective in increasing sequence coverage, as seen in nanobody discovery studies (46, 49).

### Multimerization

As mentioned earlier, two major factors that determine the final oligomeric structure of a nanobody drug are the required systemic circulatory half-life and *in vivo* antigen binding kinetics. To address these variables, multimerization of the nanobody is often performed, enabling the nanobody drug to both (i) increase its systemic retention and (ii) aid in stronger antigen binding *in vivo*. The approved drugs Ozoralizumab (approved for use in Japan) (17, 18) and Sonelokimab (113) are both nanobody trimers that incorporate a human serum albumin-binding nanobody as their central domain to increase their circulatory half-life to 18 (114) and 11 (115) days respectively. Additionally, the FDA-approved Caplacizumab is a homodimer linked by a tri-alanine repeat linker composed of two nanobodies that binds the A1 subunit of the blood glycoprotein von Willebrand factor to prevent platelet aggregation (15, 16). Furthermore, for these therapeutics, two identical antigen binding domains are incorporated into the final drug formulation, as the monomer showed significantly reduced antigen binding *in vivo* compared to the multimerized versions (116, 117).

However, the drug formulation strategy shown to be effective for currently approved nanobody drugs is not universally applicable, as there are instances where nanobody multimerization affects drug bioavailability, such as in cancer therapy when targeting solid tumors. In their monomeric state, nanobodies have been shown to successfully penetrate the tumor microenvironment better than their multimerized counterparts (118). This was seen with the anti-HER2 nanobody 2Rb17c, which showed homogenous tumor distribution of the monomeric form within minutes of injection (119), in contrast to its dimeric version and the mAb, trastuzumab, which targets the same antigen. Furthermore, when multimerizing nanobodies, especially in the context of exploring the possibility of creating a hetero-bivalent nanobody therapeutic composed of synergistic nanobodies targeting distinct epitopes on an antigen, structural information (*e.g.* X-ray crystallography or cryo-EM structures) is essential to determine drug parameters such as linker length, nanobody orientation, and nanobody formulation. Instead of experimental structural information, numerous robust computational protein modeling tools (9, 120) are available to model nanobody and antigen structures, which coupled with molecular docking programs (121, 122, 123), can be used to facilitate the best multimerization design strategy to maintain the synergistic relationship between nanobodies.

### Therapeutic Administration Routes

The structural robustness of nanobodies makes them amenable to different strategies of administration, making it possible to tailor the administration route depending on the location of the target. Of the successful nanobody drugs available, Caplacizumab (15, 16) and Ciltacabtagene autoleucel (21) are both administered intravenously, whereas Ozoralizumab (17, 18) and Envafolimab (19, 20, 124) are both administered subcutaneously (Fig. 5*B*).

More recently, orally administered nanobody drugs have been developed, which carry numerous advantages over other delivery systems including long-term stability, low production costs, and ease of administration for patients (125, 126). The primary site of drug absorption for these drugs is the gastrointestinal tract, making them particularly suitable for diseases of the gut as exemplified by the three monomeric nanobody-based drugs V565 (127, 128, 129), LMN-101 (77), and ARP1/VHH batch 203,027 (76), which have been developed as oral therapeutics to treat Crohn’s disease, traveler’s diarrhea, and rotavirus infection respectively. Notably, the delivery of LMN-101—packaged as capsules—incorporates the emerging technology of nanobody expression in the edible cyanobacterium spirulina (130), which releases the nanobody upon digestion of the cyanobacterium. The small size and low structural complexity of nanobodies readily enables the application of this technique, where orally available nanobody therapeutics could become a powerful tool in distributing drugs to developing nations, especially for the treatment of gastrointestinal diseases.

Finally, an emerging administration route that is being explored for the treatment of respiratory infections is *via* an intranasal or inhalable route, bypassing the circulatory system to actively target the lungs. These two administration routes require robust biophysical properties of the drug to withstand the formulation required for inhalable or intranasal use (131), which nanobodies can fulfill. This strategy aims to focus the nanobodies on the site of the respiratory infection to increase drug uptake and efficacy, as drugs destined for the lungs delivered systemically have shown decreased bioavailability (132, 133). In one study, a nanobody trimer treating mice infected with SARS-CoV-2 administered intranasally resulted in a faster recovery time for the animals over the same dosage administered intravenously (132). Even for classical mAbs, a mAb destined for the lung given intravenously for the treatment of asthma showed only 0.2% of the mAb reaching the lungs (134). Recently, inhalable formulations of nanobodies have been tested as a treatment option for respiratory syncytial virus (135) and SARS-CoV-2 (136, 137) infection, which could dramatically increase the accessibility of such treatments, due to their relatively easy mode of administration.

## Perspectives

Advances in techniques for large-scale nanobody generation and characterization have further spurred the already rapid growth of this class of antibodies in diverse areas of research. With multiple nanobody-based drugs coming into active clinical use in recent years, interest in the therapeutic applications of nanobodies has become particularly robust. As dramatically more nanobodies are developed and methodologies gain wider adoption, the advantages of extensive nanobody repertoires have also become increasingly clear. Nanobodies can cover greater epitope space than typical mAbs, allowing them to target difficult antigen targets, and complementary binders are capable of potent synergistic effects. Nanobodies are thus ideal candidates for combinatorial use and multimerization, along with their general flexibility as single domains suitable for wider functionalization. Many investigators are taking advantage of these uniquely favorable properties to explore new protein-targeted approaches in proteomic and cell biology research, and to expand the territory of antibody-based clinical reagents. Furthermore, the high quality and quantity of work in developing nanobodies targeting SARS-CoV-2 virus and other infectious disease agents underscores the versatility and robustness of nanobodies in the context of diagnostic and therapeutic development. Consequently, nanobodies could prove especially powerful tools in the control, treatment, and prevention of future infectious disease outbreaks, especially viral—an important strategy for the global goal of pandemic preparedness.

## Data Availability

All supporting data are provided within the manuscript, supplementary data and supplementary tables.

## Conflict of interests

The authors declare that they have no conflicts of interest with the contents of this article.
